# A Systematical Evaluation of the Crystallographic Orientation Relationship between MC Precipitates and Ferrite Matrix in HSLA Steels

**DOI:** 10.3390/ma15113967

**Published:** 2022-06-02

**Authors:** Xiaolin Li, Jiawei Yang, Yating Li, Linxi Liu, Chi Jin, Xiangyu Gao, Xiangtao Deng, Zhaodong Wang

**Affiliations:** 1 State Key Laboratory of Solidification Processing, Center of Advanced Lubrication and Seal Materials, Northwestern Polytechnical University, Xi’an 710072, China; 2019301036@mail.nwpu.edu.cn (J.Y.); liyating@mail.nwpu.edu.cn (Y.L.); linxiliu@mail.nwpu.edu.cn (L.L.); jinchi@mail.nwpu.edu.cn (C.J.); 2 Analysis and Testing Center, Northwestern Polytechnical University, Xi’an 710072, China; tsgxxiangyugao@nwpu.edu.cn; 3 State Key Laboratory of Rolling and Automation, Northeastern University, Shenyang 110819, China; 1310155@neu.edu.cn

**Keywords:** crystallographic, ultra-fast cooling, precipitation behavior, orientation relationship, nano-precipitates

## Abstract

Here we systematically investigate the crystallographic orientation relationship (OR) between MC-type precipitates (M, metal; C, carbon) and ferrite matrix in the Ti-Mo microalloyed steel with different processing. In the specimens without austenite deformation, the interphase precipitation can be obtained, and the precipitates obey Baker–Nutting (BN) OR with ferrite matrix. By contrast, in the specimens with austenite deformation, the supersaturated precipitates were formed in ferrite grains, which can obey BN, Nishiyama–Wasserman (NW), Kurdjumov–Sachs (KS) and Pitsch (P) ORs simultaneously. The cooling rate after austenite deformation can influence the OR between carbides and ferrite in the MC/ferrite system. At the cooling rate of 80 °C/s, carbides and ferrite can roughly satisfy these OR with the deviation ≥ 10°, while at the cooling rate of 20 °C/s, carbides and ferrite can strictly obey the specific OR. The energy accumulated in the deformation process and maintained in the fast-cooling process (80 °C/s) can offset the formation energy of the carbides. Thus, the carbides formed in the specimen with the cooling rate of 80 °C/s do not strictly satisfy the specific ORs to meet the rule of lowest energy, and then deviate by a small angle based on the specific ORs.

## 1. Introduction

High strength low-alloy (HSLA) steels have attracted extensive attention recently for their remarkable high strength and high toughness, and have been widely used in buildings, bridges, and ships [1,2,3,4]. These steels derive their high strength from a combination of grain refinement strengthening, solid-solution strengthening, dislocation strengthening, and precipitation hardening [5,6,7]. Among these strengthening methods, precipitation strengthening is the only mechanism that can improve strength without sacrificing ductility besides grain refinement strengthening [8,9]. In HSLA steels, Nb, V, Ti, and Mo alloys are typically added to form MC-type carbides with abundant C available in ferrite to strengthen steel. Funakawa [10] developed Ti-Mo bearing HSLA steel with tensile strengths of 780 MPa and excellent formability. The microstructure of the steel consists of ferrite matrix with nanometer-sized carbides, and the precipitation hardening has been estimated as ~300 MPa.

According to the Ashby–Orowan equation $\Delta \sigma =10.8\sqrt{f}/d\ln(1630d)$, it can be concluded that precipitate size and volume fraction are the two main contributors to precipitation strengthening. Thus, controlling precipitate formed at nanoscale size and high-density is essential [11,12,13]. All MC-type carbides in steels were developed based on the nucleation theory. According to the nucleation theory, the nucleation rate is negatively exponentially related to the critical energy. The nucleation rate, critical energy, and coarsening rate can be calculated by Equations (1)–(3):

(1) $$\dot{N}\propto \exp\frac{-\Delta G*}{kT}$$

(2) $$\Delta G*=\frac{16\pi \gamma^{3}}{3(\Delta G_{v}+\Delta G_{E})}$$

(3) $$r_{t}^{n}-r_{0}^{n}=\frac{k}{RT}V_{m}^{2}CD\gamma t$$

where $\dot{N}$, ΔG*, $\Delta G_{v}$, $\Delta G_{E}$, γ, *r*_0_ and *r_t_* represent the nucleation rate, driving force, elastic strain energy, interfacial energy, and original and final precipitate size, respectively.

Based on these equations, we can conclude that nucleation rate, thus the number density can be increased by three methods: (i) reducing the interfacial and elastic strain energy; (ii) increasing the nucleation driving force; and (iii) increasing the sites for nucleation. The nucleation sites and driving force can be controlled by adjusting the processing parameters. By contrast, the interfacial energy and elastic energy can be reduced by adjusting the chemistry of precipitate and matrix. This method has been successfully used in different steels. Jiang et al. [14] created a new class of steels strengthened by high-density dispersed coherent nanoscale Ni(Al, Fe) precipitates by adding Al, Mo, and Nb to minimize the interfacial energy. Funakawa et al. [10] reported that Mo substitution for Ti in TiC reduced the interfacial energy between TiC and ferrite in low-carbon steels. Adjusting the atoms’ distribution in the grain boundary, and thus the OR between precipitates and matrix, is also an effective method. Our previous research [15] has found a new OR MC/ferrite system in Nb-Ti microalloyed steel with lower interfacial energy, and the number density is relatively higher compared with that obey other ORs. 

The ORs between MC and ferrite depend on the precipitation reaction mode and the processing. In austenite, the precipitates obey cube-on-cube OR with austenite, and in ferrite the precipitates obey the BN or NW OR with ferrite. The OR between precipitates and matrix can affect the preferred growth direction, nucleation, and coarsening rate, and then directly affect precipitate shape and mechanical properties [16,17,18]. Therefore, the determination of OR has guiding significance, in theory, for understanding the essence of microstructure, such as crystal growth and phase transformation. Moreover, the study of OR is also of great value for the development of materials and the rational selection of processing technology. Extensive research [17,19,20,21,22] has been conducted on the OR between precipitates and ferrite matrix in Ti-bearing experimental steels, but it is far from industrialization because it has no deformation and industrial conditions. 

In this study, we focus on the studying the OR between precipitates and ferrite in Ti-Mo steel in different processing, and systematically investigate the effect of austenite deformation and cooling rate after deformation on the OR in MC/ferrite system, and establish the relationship between OR and processing. 

## 2. Materials and Experimental Procedure

**Figure 1:**
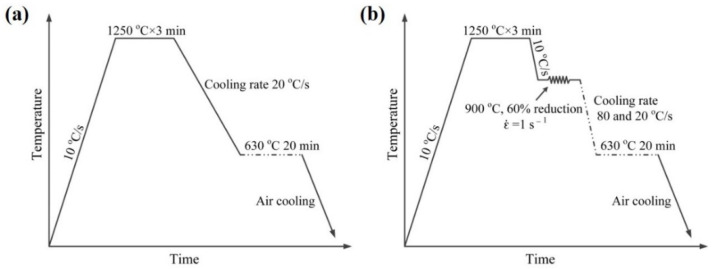
Schematic diagrams showing the simulation process (**a**) I, (**b**) II.

The chemical composition of the experimental steel was Fe-0.09C-1.05Mn-0.25Si-0.03V-0.025Nb-0.011Ti-0.26Mo (wt.%). The steel was prepared by vacuum melting and cast into ingot of thickness ~100 mm. The ingots were homogenized at 1473 K (1200 °C) for 1 h and then hot-rolled into steel plate of 12 mm thickness via seven passes on the Φ450 mm trial rolling mill. The specimens were prepared from the mid-thickness position along the rolling direction and machined into 8 mm diameter cylindrical rods of 15 mm length. The cylinder specimens were used to simulate the static isothermal process and dynamic isothermal process with different cooling rate after austenite deformation by MMS-300 thermo-mechanical simulator. Two processes have been simulated: (I) isothermal holding process without austenite deformation and (II) isothermal holding process after austenite deformation with different cooling rates. For process I, the specimen was austenitized at 1250 °C for 3 min to dissolve all the carbides, and then cooled to 630 °C at a cooling rate of 20 °C/s and kept at held for 20 min respectively, and then air cooled to room temperature, as shown in Figure 1a. For process II, the specimens were austenitized at 1250 °C for 3 min, deformed at 900 °C with a reduction of 60%, cooled to 630 °C at a cooling rate of 80 °C/s and 20 °C/s and kept at this temperature for 20 min, and then air-cooled to room temperature, as shown in Figure 1b.

TEM specimens were mechanically ground to 50 μm, and disks of 3 mm diameter were punched from the thin foils and electrolytically jet polished in a solution of 9 vol.% perchloric acid in ethanol at −20 °C with a Struers Tenupol 5 twin-jet electropolisher at a voltage of 30 V. TEM experiments, including selected area electron diffraction (SAED) and high-resolution TEM (HRTEM) were carried out using an FEI Talos F200X microscope operating at 200 kV to characterize the morphology, distribution, and crystallography of precipitates and matrix. 

## 3. Results and Discussions

### 3.1. Summary of Common ORs in MC/Ferrite System

The ferrite matrix possesses the body-centered cubic (bcc) structure with the lattice parameter of 0.286 nm, and the MX (M, one or more of Ti, Nb, V, and Mo; X, C and N) precipitates formed in steel are face centered cubic (fcc) structure. The lattice parameters of the MC-type precipitate are in the range of 0.41–0.45 nm. The precipitates can obey BN, NW, and KS ORs, and the less common P OR with ferrite matrix, which has been reported in the ferrite-austenite system during the solidification process [23]. The typical characteristics of the four ORs are shown in Table 1, including the parallel alignments, number of variant, close-packed components, and the smallest misfit components. The description of the ORs usually described by a parallel alignment of planes and a set of directions within the parallel planes.

**Figure 2:**
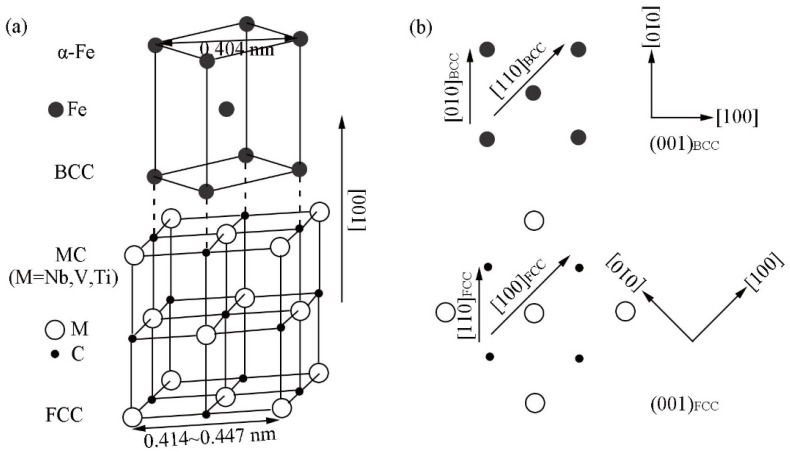
(**a**) Three-dimensional schematic diagram of BN OR; (**b**) The schematic diagram of atom distribution in (100)_MC_//(100)_α_ of BN OR.

Among all the common ORs between precipitate and ferrite, the most common one is the BN OR, namely ${\left\{001\right\}}_{\mathrm{MC}}{//\{001\}}_{\alpha }$ and ${<110>}_{\mathrm{MC}}{//<100>}_{\alpha }$. Since $\sqrt{2}a_{\mathrm{ferrite}}$ (0.287 nm) is close to the lattice constant of MC (0.41~0.45 nm), ${<100>}_{\mathrm{MC}}$ and $<110{>}_{\alpha }$ can be well matched in both directions and satisfy the minimum degree of mismatch in the initial stage of nucleation, as shown in Figure 2. 

**Figure 3:**
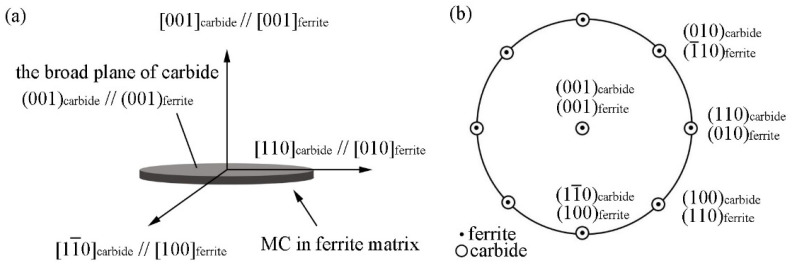
Illustration of OR between MC precipitates and ferrite matrix under BN 1a (**a**) and corresponding stereographic projection (**b**).

Figure 3 shows the illustration of the OR between MC precipitates and ferrite matrix obey BN 1a (${\left(001\right)}_{\mathrm{MC}}{//(001)}_{\alpha }$ and ${\left[110\right]}_{\mathrm{MC}}//{\left[010\right]}_{\alpha }$) and the corresponding stereographic projection. There are two equivalent <110> crystal orientations in the ${\left(001\right)}_{\mathrm{MC}}$ plane, [110] and $\left[1\bar{1}0\right]$, as shown in Figure 3a. These two crystal directions are perpendicular and $\left[1\bar{1}0\right]$ can be obtained by rotating ${\left[110\right]}_{\mathrm{MC}}$ around the normal direction of ${\left(001\right)}_{\mathrm{MC}}$ by 90°. Therefore, there are three variants in BN OR, as listed in Table 2. In previous studies, it was mentioned that three different variants of the BN OR were observed in the same ferrite grain [24].

**Figure 4:**
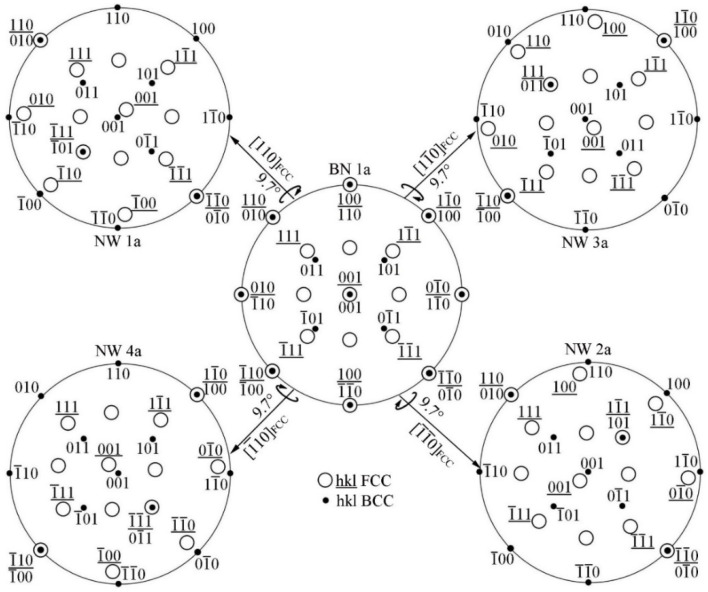
Schematic diagram showing BN 1a transferred to four NW OR variants by rotation axis.

Comparing the four ORs listed in Table 1, it is found that there is a certain correlation; that is, winding a small crystal plane angle rotation can realize mutual conversion. The conversion relationship will be discussed in detail in following. Figure 4 shows the conversion of BN 1a OR to NW OR. The BN OR has four-fold symmetry relationship, so one variant can be converted into four variants of the NW OR. NW 1a can be obtained by rotating BN 1a around ${\left[110\right]}_{\mathrm{MC}}$ by 9.74° counterclockwise so that ${\left(\bar{1}11\right)}_{\mathrm{MC}}$ and ${\left(\bar{1}01\right)}_{\alpha }$ are parallel. NW 2a can be obtained by rotating BN 1a around ${\left[\bar{1}\bar{1}0\right]}_{\mathrm{MC}}$ by 9.74° counterclockwise so that ${\left(1\bar{1}1\right)}_{\mathrm{MC}}$ and ${\left(101\right)}_{\alpha }$ are parallel; NW 3a can be obtained by rotating BN 1a around ${\left[1\bar{1}0\right]}_{\mathrm{MC}}$9.74° counterclockwise so that ${\left(111\right)}_{\mathrm{MC}}$ and ${\left(011\right)}_{\alpha }$ are parallel; NW 4a can be obtained by rotating around ${\left[\bar{1}10\right]}_{\mathrm{MC}}$ by 9.74° counterclockwise so that ${\left(\bar{1}\bar{1}1\right)}_{\mathrm{MC}}$ and ${\left(0\bar{1}1\right)}_{\alpha }$ are parallel. Because there are three variants of the BN OR, it can be inferred that NW has 12 variants. Both the NW OR and the BN OR satisfy $<100{>}_{\mathrm{MC}}//<110{>}_{\alpha }$, while the NW OR also satisfies the close-packed plane parallel alignment ${\left\{111\right\}}_{\mathrm{MC}}{//\{110\}}_{\alpha }$.

The P OR was first discovered during the transformation of austenite to martensite in microalloyed steel with nitrogen addition, and was also observed in precipitates and matrix systems. For example, it was observed in Cr-rich precipitates and Cu matrix in Cu-Cr alloy, Cr-rich precipitates and Ni matrix in Ni-Cr alloy, α-Fe precipitates and Cu matrix, γ-Fe_4_N precipitates with fcc structure and α-Fe matrix. This OR is ${\left\{200\right\}}_{\mathrm{FCC}}{//\{110\}}_{\mathrm{BCC}}$ and ${<111>}_{\mathrm{FCC}}{//<110>}_{\mathrm{BCC}}$, has quadratic symmetry, and satisfies parallel close packing directions and low misfit crystal plane matching.

**Figure 5:**
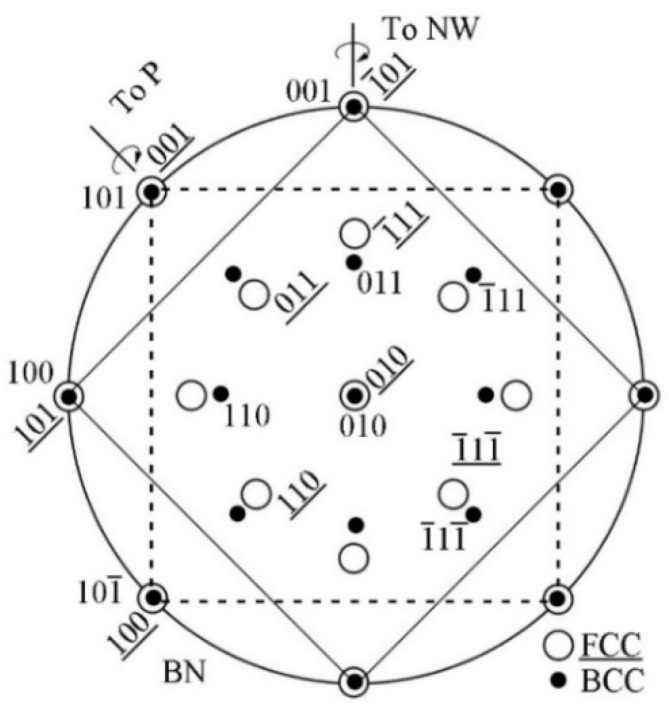
Schematic diagram showing the BN OR transformed to NW and P ORs.

The BN OR satisfies ${\left(010\right)}_{\mathrm{MC}}{//(010)}_{\alpha }$, ${\left(\bar{1}01\right)}_{\mathrm{MC}}{//(001)}_{\alpha }$ and ${\left(001\right)}_{\mathrm{MC}}{//(101)}_{\alpha }$. The NW OR can be obtained by rotating 9.7° around ${\left[\bar{1}01\right]}_{\mathrm{MC}}$. The P OR can be obtained by rotating around 9.7° around the ${\left[101\right]}_{\mathrm{MC}}$, as shown in Figure 5. Although there are differences in the crystallographic relationship between the P OR and NW OR, the symmetry, minimum lattice misfit planes, and the rotation angle plane are the same. There are 12 variants of P ORs.

**Figure 6:**
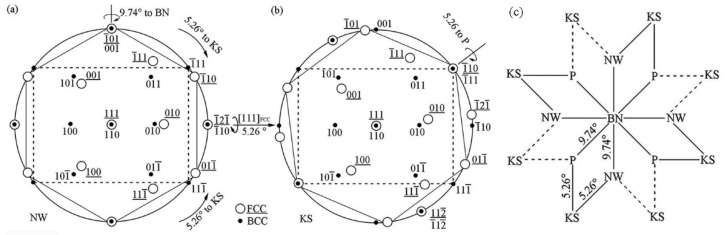
Schematic diagram illustrating the stereographic projection of NW OR (**a**) and KS OR (**b**), and the symmetry diagram pointing out the interrelationships between different variants of the four main orientation re1ationships in BCC/FCC systems (**c**).

The typical feature of the KS OR is that the ${\left\{111\right\}}_{\mathrm{FCC}}$ close-packed planes are parallel to the ${\left\{110\right\}}_{\mathrm{BCC}}$ close-packed planes; the close-packed direction <110> in the ${\left\{111\right\}}_{\mathrm{FCC}}$ planes is parallel to that <111> in ${\left\{110\right\}}_{\mathrm{BCC}}$. The common characteristic between KS and NW ORs is the parallel close-packed planes, but the KS OR simultaneously satisfies the condition that close-packed directions in close-packed planes are parallel. Figure 6a,b exhibits the stereographic projection of NW and KS ORs. The NW OR can be obtained by rotating KS OR around ${\left[111\right]}_{\mathrm{MC}}$ counterclockwise 5.26°.

Through the analysis of the four ORs, it can be concluded that the P OR and the NW OR can be converted to each other through the BN OR and KS OR along a certain crystal plane with a small angle rotation, as shown in Figure 6c.

### 3.2. Precipitation Behavior in the Specimen with and without Deformation

**Figure 7:**
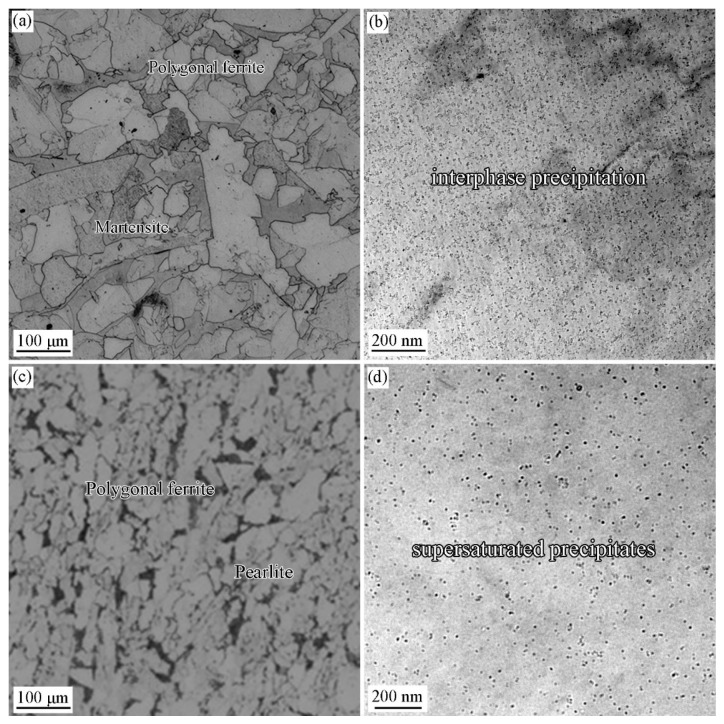
Optical microstructures (**a**) and TEM images (**b**) of Ti-Nb steel isothermally held at 660 °C for 20 min without austenite deformation; Optical microstructures (**c**) and TEM images (**d**) of Ti-Nb steel isothermally held at 660 °C for 20 min after austenite deformation.

Figure 7 shows the optical images and TEM images showing the microstructure and precipitation behavior of Ti-Mo steel isothermal at 660 °C for 20 min with and without deformation at 900 °C. The grains were refined by the deformation to a large extent by comparing the grain size of two specimens. The grain size is 48 μm in the specimen without deformation, and is 6.8 μm for the specimen with deformation (Figure 7a,c). Figure 7b,d shows the morphology of precipitates with and without deformation and no deformation conditions respectively. It can be seen that interphase precipitation dominates in the specimens without deformation, while after deformation precipitates are randomly distributed, that is supersaturated precipitates. Heavy deformation in the non-recrystallized zone of austenite can greatly promote the formation of ferrite and pearlite, and the diffusion of solute elements is only related to temperature [25,26,27]. The interphase precipitation formation needs to balance the diffusion rate of solute atoms and the migration capabilities of γ/α phase boundaries.

**Figure 8:**
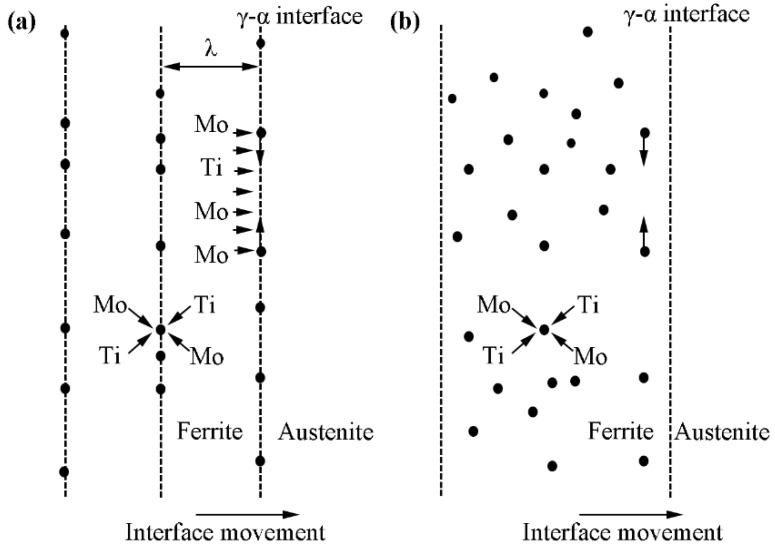
Schematic diagrams showing the relationship between precipitation morphology and the velocity of γ/α interface (**a**) interphase precipitation, (**b**) supersaturated precipitates.

Figure 8 presents the schematic diagram illustrating the relationship between precipitation morphology and the moving velocity of γ/α interface. Figure 8a shows the schematic diagram of interphase precipitation formation. When interface moving speed matches the diffusion rate, it can facilitate the interphase precipitation formation. Figure 8b shows the schematic diagram of supersaturated precipitation formation. The heavy deformation in the non-recrystallization zone increases the moving speed of the γ/α interface, and the diffusion speed of solute elements cannot match the moving speed of the interface, then the solute alloys will keep in the transformed ferrite matrix [26,27]. Since the solid solubility of microalloyed elements in ferrite is two orders of magnitude smaller than that in austenite, these supersaturated microalloyed elements will precipitate in the form of carbides in the subsequent isothermal process.

### 3.3. ORs between MC and Ferrite in Specimen with Process I

**Figure 9:**
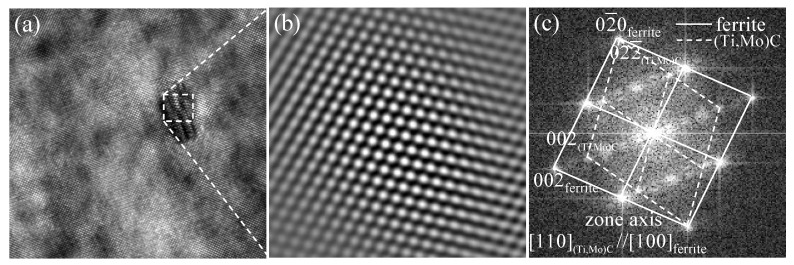
HRTEM image of precipitate and the OR between the precipitates and ferrite matrix in the Ti-Mo steel isothermal holding at 660 °C for 20 min (**a**) HRTEM image; (**b**) iFFT diffractogram; (**c**) FFT diffractogram.

Figure 9 shows the HRTEM image and the corresponding analysis of interphase precipitate formed in the specimens without austenite deformation. As shown in Figure 9a, the carbide is so fine that it lies within the foil thickness. This leads to the development of a Moiré fringe contrast due to the overlapping carbide and ferrite lattices. Figure 9b shows the corresponding fast Fourier transformed (FFT) diffractogram, which proves that that carbide has a fcc crystal structure. The OR between carbide and ferrite matrix has been identified by the FFT diffractogram, as illustrated in Figure 9c. Extra diffraction spots are related to iron oxide on the surface and double diffraction from oxide and ferrite. The orientation relationship of carbide with respect to ferrite matrix is ${\left[110\right]}_{\mathrm{MC}}//{\left[100\right]}_{\alpha }$ and ${\left(02\bar{2}\right)}_{\mathrm{MC}}{//(0\bar{2}0)}_{\alpha }$. Three carbides were analyzed, exhibiting the same variant of BN OR with the ferrite matrix. The preferred variant can maintain the smallest angle between the interface of ferrite and the carbide broad., and it is preferable to other variants during the interphase precipitation.

### 3.4. ORs between MC and Ferrite in Specimen with Process II

**Figure 10:**
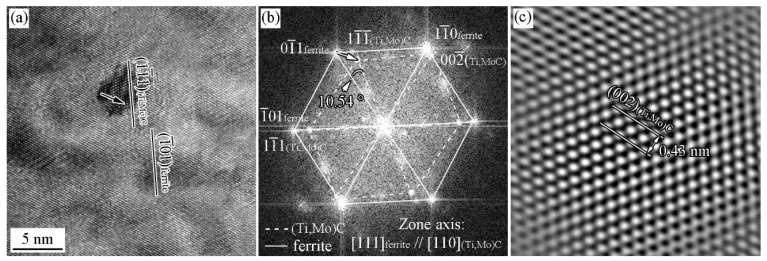
HRTEM image of precipitates and the ORs between the precipitates and ferrite matrix in the Ti-Mo steel produced by process II with cooling rate 20 °C/s (**a**) HRTEM image; (**b**) FFT diffractogram; (**c**) IFFT diffractogram.

**Figure 11:**
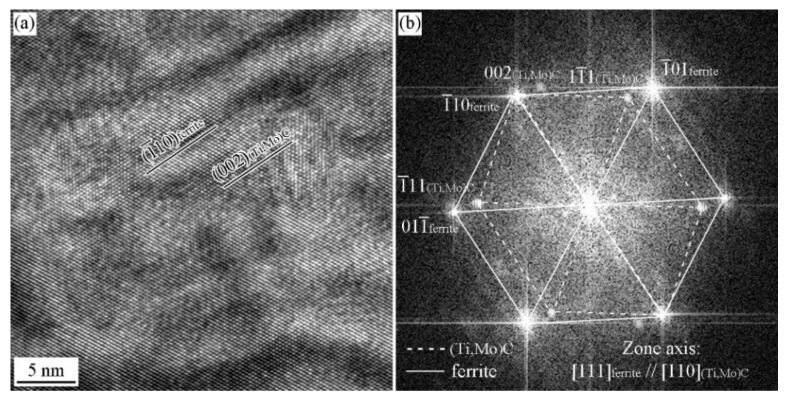
HRTEM image of precipitates and the ORs between the precipitates and ferrite matrix in the Ti-Mo steel produced by process II with the cooling rate of 20 °C/s (**a**) HRTEM image; (**b**) FFT diffractogram.

Figure 10 and Figure 11 show the HRTEM image of precipitates and the ORs between the precipitates and ferrite matrix in the Ti-Mo steel produced by process II with the cooling rate of 20 °C/s. The carbide shown in Figure 10 strictly satisfies the ${\left(111\right)}_{\mathrm{MC}}{//(101)}_{\alpha }$ and ${\left[110\right]}_{\mathrm{MC}}//{\left[111\right]}_{\alpha }$ with ferrite matrix, which is a variant of KS OR with the ferrite (Figure 10b). Figure 10c shows iFFT of the carbide. The lattice parameter of the carbide is 0.43 nm based on the measurement of the interplanar spacing of (002). The carbide in Figure 11a obeys ${\left(002\right)}_{\mathrm{MC}}{//(\bar{1}10)}_{\alpha }$ and ${\left[110\right]}_{\mathrm{MC}}//{\left[111\right]}_{\alpha }$ with ferrite matrix, that is P OR, which was analyzed by the analysis of FFT diffractogram in Figure 11b.

**Figure 12:**
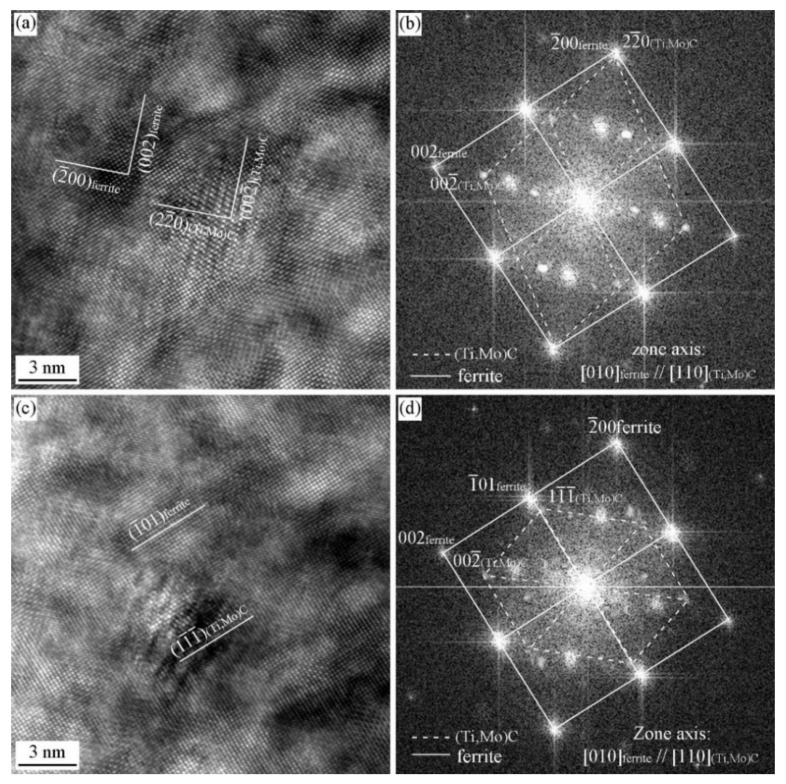
HRTEM image of precipitates and the ORs between the precipitates and ferrite matrix in the Ti-Mo steel produced by process II with the cooling rate of 20 °C/s (**a**,**c**) HRTEM image; (**b**,**d**) FFT diffractogram.

Except for KS and P OR observed in [111]_α_ zone axis, BN and NW ORs observed in [111]_α_ zone axis have also been obtained. Figure 12a,b shows the HRTEM image of the carbide and the corresponding FFT analysis. The carbide obeys ${\left[110\right]}_{\mathrm{MC}}//{\left[010\right]}_{\alpha }$ and ${\left[2\bar{2}0\right]}_{\mathrm{MC}}{//[\bar{2}00]}_{\alpha }$ OR with ferrite matrix, that is a variant of BN OR. Figure 12c,d shows the HRTEM image of the carbide and the corresponding FFT analysis. The carbide obeys ${\left[110\right]}_{\mathrm{MC}}//{\left[010\right]}_{\alpha }$ and ${\left[1\bar{1}\bar{1}\right]}_{\mathrm{MC}}{//[\bar{1}01]}_{\alpha }$ OR with ferrite matrix, which is a variant of NW OR.

**Figure 13:**
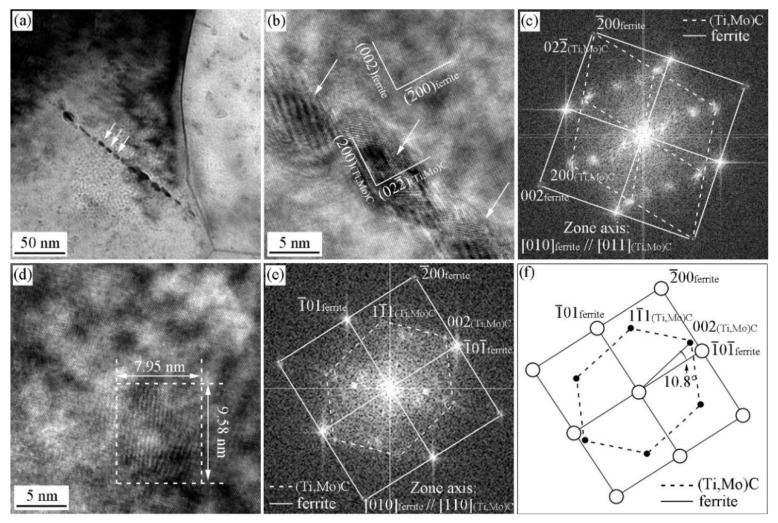
HRTEM image of precipitates and the ORs between the precipitates and ferrite matrix in the Ti-Mo steel produced by process II with the cooling rate of 80 °C/s (**a**,**b**) TEM and HRTEM images of planer carbides, and the arrow pointing the planer precipitates after grain boundaries moving; (**c**) FFT diffractogram of planer carbides; (**d**) HRTEM image of the supersaturated carbide; (**e**) FFT diffractogram; (**f**) the simulated diffraction pattern of the supersaturated carbide.

**Figure 14:**
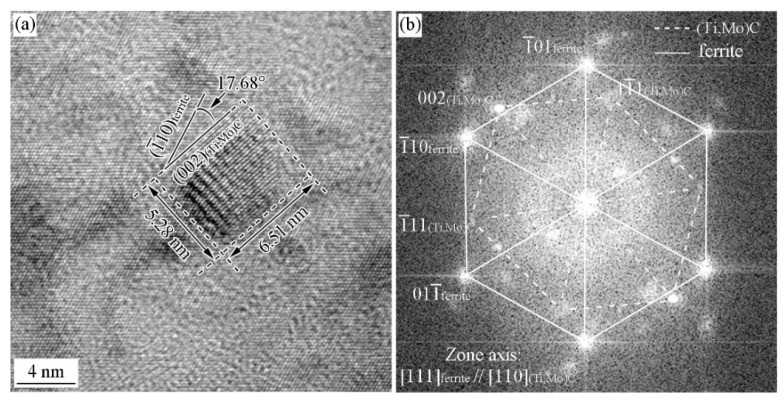
HRTEM image of precipitate and the OR between precipitate and ferrite matrix in Ti-Mo steel produced by process II with the cooling rate of 80 °C/s (**a**) HRTEM image; (**b**) FFT diffractogram.

Figure 13 and Figure 14 show the HRTEM images and the corresponding FFT diffractogram the precipitates and ferrite matrix in the Ti-Mo steel produced by process II with the cooling rate of 80 °C/s. Two type of precipitates have been obtained, planer precipitates formed in the dislocation line or sub-grain boundary and supersaturated precipitates. Figure 13a–c shows the TEM image, HRTEM image, and the corresponding FFT diffractogram of the planer precipitates. It can be seen that the precipitates obey BN OR with ferrite matrix, and the three precipitates obey the same variant of BN OR, i.e., ${\left(02\bar{2}\right)}_{\mathrm{MC}}{//(\bar{2}00)}_{a}$ and ${\left[011\right]}_{\mathrm{MC}}{//[010]}_{\alpha }$. Figure 13d–f shows HRTEM images, FFT diffractogram, and the simulated diffraction pattern of supersaturated precipitates. The carbide and ferrite matrix does not strictly obey the specific OR (BN or NW ORs), and deviates a small angle.

Figure 14 shows the HRTEM images and the FFT diffractogram of a supersaturated precipitate. The carbide and ferrite matrix doesn’t strictly obey the specific OR (KS or P ORs), and deviates a small angle. The above analysis confirm that the precipitates obtained from the Ti-Mo steel with the cooling rate of 80 °C/s after deformation cannot satisfy the specific ORs with ferrite matrix for the energy accumulated and maintained in the deformation and fast cooling process, which can be used to offset the formation energy of precipitates.

## 4. Conclusions

The precipitation behavior and orientation relationships between precipitates and ferrite formed in Ti-Mo alloys with different processing have been investigated. The effects of austenite deformation and cooling rate after austenite deformation on precipitation behavior and orientation relationship have also been analyzed. The main conclusions are as follows:

1. In the isothermal holding process without austenite deformation, interphase precipitation can be obtained, and these precipitates obey BN ORs with ferrite matrix.
2. In the isothermal holding process with austenite deformation, supersaturate precipitates have been obtained, and these precipitates can obey the BN, NW, KS, and P ORs in the same specimens.
3. With the cooling rate of 20 °C/s after austenite deformation, the precipitates and ferrite can strictly satisfy the specific OR, while at the cooling rate of 80 °C/s, precipitates and ferrite can roughly satisfy these OR with the deviation ~10°.

## Figures and Tables

**Table 1:** Summary of the known orientation relationship between second phases and ferrite matrix.

Name	Orientation Relationship	Variant	Close-Packed Components	Smallest Misfit
BN	{001}_MC_//{001}_α_ <110> _MC_//<100>_α_	3	None	{011}_MC_//{001}_α_
P	{001}_MC_//{101}_α_ <$\bar{1}$10> _MC_//<$\bar{1}$11>_α_	12	CP directions	{002}_MC_//{011}_α_
NW	{111}_MC_//{110}α <$\bar{1}$01> _MC_//<001>_α_	12	CP planes	{111}_MC_//{110}_α_
KS	{111}_MC_//{110}α <$\bar{1}$10> _MC_//<$\bar{1}$11>_α_	24	CP planes/directions	{111}_MC_//{110}_α_

**Table 2:** Three variants of BN ORs between carbide and ferrite.

BN Variant	Parallel Plane	Parallel Direction	Parallel Direction
BN 1a	(001)_MC_//(001)_α_	[110]_MC_//[010]_α_	[100]_MC_//[110]_α_
BN 1b	(001)_MC_//(001)_α_	[1$\bar{1}$0]_MC_//[010]_α_	[0$\bar{1}$0]_MC_//[110]_α_
BN 2a	(100)_MC_//(001)_α_	[011]_MC_//[010]_α_	[010]_MC_//[110]_α_
BN 2b	(100)_MC_//(001)_α_	[01$\bar{1}$]_MC_//[010]_α_	[00$\bar{1}$]_MC_//[110]_α_
BN 3a	(010)_MC_//(001)_α_	[101]_MC_//[010]_α_	[001]_MC_//[110]_α_
BN 3b	(010)_MC_//(001)_α_	[10$\bar{1}$]_MC_//[010]_α_	[100]_MC_//[110]_α_
